# Kidney stone analysis: “Give me your stone, I will tell you who you are!”

**DOI:** 10.1007/s00345-014-1444-9

**Published:** 2014-12-03

**Authors:** Jonathan Cloutier, Luca Villa, Olivier Traxer, Michel Daudon

**Affiliations:** 1 Urology Department, Tenon University Hospital, 4 rue de la Chine, 75970 Paris Cedex 20, France; 2 Service des Explorations Fonctionnelles, Tenon University Hospital, 4 rue de la Chine, 75970 Paris Cedex 20, France

**Keywords:** Urinary calculi, Stone morphology, Etiology, Stone analysis, Calcium oxalate, Calcium phosphate

## Abstract

**Introduction:**

Stone analysis is an important part in the evaluation of patients having stone disease. This could orientate the physician toward particular etiologies.

**Material and methods:**

Chemical and physical methods are both used for analysis. Unfortunately, chemical methods often are inadequate to analyze accurately urinary calculi and could fail to detect some elements into the stone. Physical methods, in counterpart, are becoming more and more used in high-volume laboratories. The present manuscript will provide a review on analytic methods, and review all the information that should be included into an appropriate morpho-constitutional analysis.

**Conclusion:**

This report can supply an excellent summarization of the stone morphology and give the opportunity to find specific metabolic disorders and different lithogenic process into the same stone. Here, specific chemical types with their different crystalline phases are shown in connection with their different etiologies involved.

## Introduction

Over the last decades, urolithiasis has been increasingly diagnosed, and nowadays, it affects roughly 10 % of the Western countries population [1–5]. Dramatic changes in dietary habits including a high protein and salt intake, and more recently a high consumption of carbonated beverage rich in fructose represent one of the major causes of an increased incidence of calcium oxalate renal stone, which now represents the most frequently diagnosed type of stone. [6–8]. However, several other factors may be implied in stone formation. Indeed, more than 100 chemical components have been identified in urinary calculi [9], and more than 100 different etiologies may be involved in stone formation. Among analytical methods for identifying the stone components, chemical and physical methods can be used. However, despite their low cost, chemical methods are often inadequate for accurately analyzing urinary calculi. They are unsuccessful to identify rare purine stones resulting from genetic disorders such as 2,8-dihydroxyadenine [10–12] or drug-induced calculi [13–15]. Moreover, they are unable to quantify the respective amount of each element in mixed stones and to differentiate accurately between the various crystalline phases of calcium oxalate or calcium phosphate that are related to very different biochemical and pathophysiological conditions [16–18].

## Physical analytic methods

Among physical methods, X-ray diffraction (XRD) and Fourier transform infrared spectroscopy (FTIR) are currently used for stone analysis. They identify each component and provide semi-quantitative evaluation of their proportions within the stone.

**Fig. 1:**
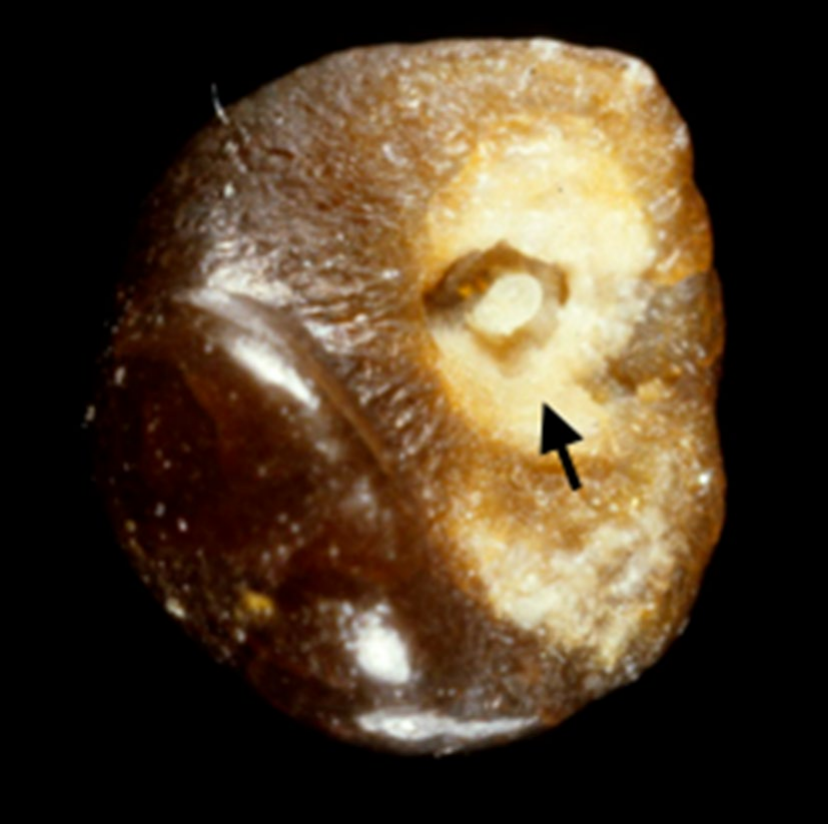
Whewellite stone initiated from a carbapatite Randall’s plaque (*arrow*)

**Fig. 2:**
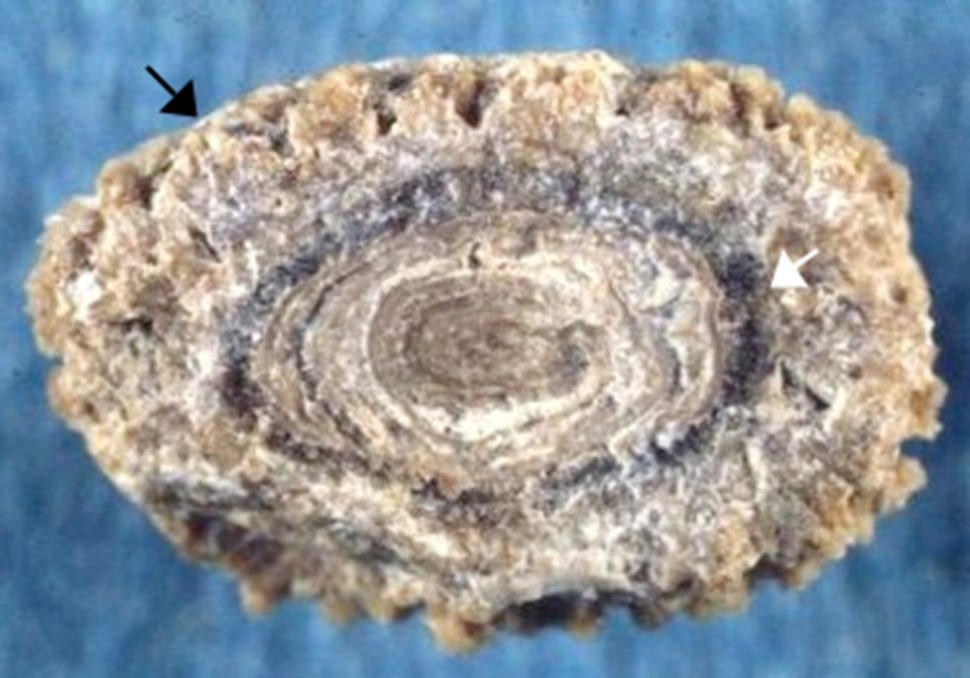
Kidney stone from a child aged 9 years old. Examination of the stone section shows a mixed stone initiated by crystallization of ammonium hydrogen urate as a result of chronic diarrhea related to bowel infection while the child ate a vegetable diet providing insufficient protein and phosphorus intake. Ammonium urate was secondly covered by whewellite (*white arrow*) as a consequence of oxalate-rich diet and low water intake. The further coverage by a mixture of weddellite and carbapatite (*black arrow*) was the consequence of changes in life style resulting in an easy access to dairy products. The child developed hypercalciuria of dietary origin

**Fig. 3:**
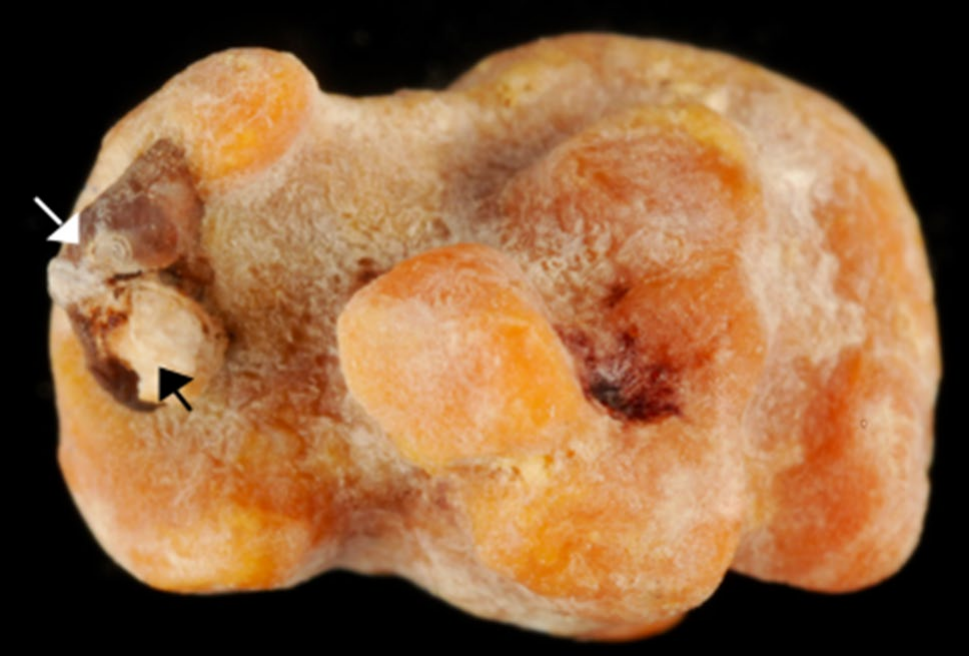
Uric acid kidney stone in a man aged 58 years old. The patient had a BMI above 30 kg/m^2^ and suffered a type 2 diabetes mellitus and hypertension. Uric acid was the consequence of metabolic syndrome and diabetes. However, stone analysis provided evidence that uric acid was secondly deposited on a whewellite stone (*white arrow*) and that the first step of stone formation was a carbapatite Randall’s plaque (*black arrow*), suggesting the stone was initiated for a long time

**Fig. 4:**
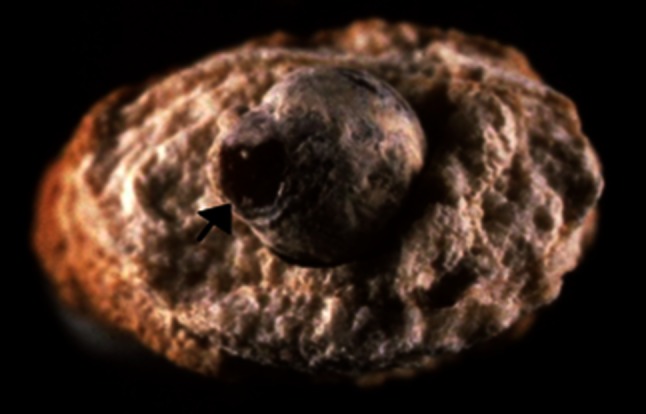
Section of a stone presumably related to urinary tract infection. In fact, while the peripheral layers are made of a mixture of carbapatite and struvite as a consequence of chronic UTI, the core of the stone is made of pure whewellite, suggesting that metabolic factors are first involved in the stone process. Of note, the morphology of the initial whewellite stone shows a papillary imprint (*arrow*) which is highly suggestive of heterogeneous nucleation from a Randall’s plaque (not visible)

These methods are able to identify non-calcium stones such as cystine, 2,8-dihydroxyadenine, xanthine, uric acid, urates, methyl-1 uric acid, struvite, proteins, lipids or drugs, as well as calcium oxalate (CaOx) and/or calcium phosphate (CaPh) stones. Because stones may remain several months or years in the urinary tract, they contain commonly (94 % in our experience) several components [19]. An accurate identification of minor components with their location in the stone is clinically relevant to assess environmental factors involved or to explain the lithogenic process (for example crystallization of CaOx from a CaPh Randall’s plaque). Moreover, it could highlight marked changes in conditions with outbreak of new lithogenic process such as primary hyperparathyroidism, type 2 mellitus diabetes or urinary tract infection by urea-splitting bacteria (Figs. 1, 2, 3, 4).

**Fig. 5:**
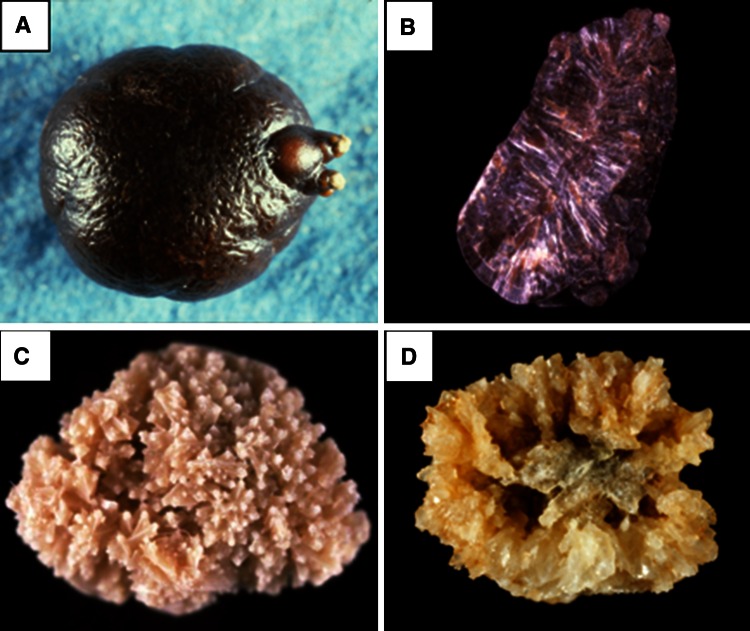
Common stones made of calcium oxalate. Stone morphology is very different according to the crystalline phase: Calcium oxalate monohydrate corresponding to the stone subtype Ia (**a** surface; **b** section). Calcium oxalate dihydrate corresponding to the stone subtype IIa (**c** surface; **d** section)

Physical methods provide information on crystalline phases of the same chemical species that may imply different lithogenic conditions: for example, whewellite (CaOx monohydrate, COM) and weddellite (CaOx dihydrate, COD) among CaOx stones; carbapatite, brushite, or whitlockite among CaPh stones. A similar composition, for example CaOx, may be the result of a variety of lithogenic processes, including diet imbalance, low diuresis, genetic or acquired diseases [9]. It is the same for crystalline phases: COM stones may correspond to hyperoxaluric states related to very different etiopathogenic conditions, such as primary hyperoxaluria, enteric hyperoxaluria, or idiopathic CaOx nephrolithiasis [16, 20]. In contrast, COD stones are clearly related to hypercalciuria in a very high proportion of cases [16, 20, 21]. The corresponding stones exhibit distinct morphology easily identified in both surface and section (Fig. 5). Finally, the initial nucleation process could be related to another mechanism (e.g., Randall’s plaque) than the factors responsible for the subsequent stone growth. All these considerations raise the importance that the stone analysis should provide information on the stone morphology, chemical composition and crystalline phases, as well as their location within the stone.

Only physical methods can identify such a diversity of components. For this purpose, several techniques were proposed in routine practice [22], including X-ray powder diffraction [23], infrared spectroscopy [24], Raman spectroscopy [25, 26], scanning electron microscopy [27] or thermal analysis [28] and also stereomicroscopy for stone morphology [9, 20]. Nowadays, infrared spectroscopy is extensively used for more than 300,000 stone analyses per year over the world. One limitation for some centers to use this technology can be the cost associated with the equipment, infrared spectroscopy is the less expensive, and X-ray powder diffraction and scanning electron microscopy are the most expensive.

## What information from stone analysis could be clinically relevant?

A stone might be the first manifestation of numerous pathologies and metabolic disorders. The objective of stone analysis is to collect all relevant information from the stone helping the physician to establish the cause(s) of stone formation and growth. For that purpose, physicians may investigate blood and urine biochemistry of each stone former in order to identify metabolic disorders able to provide accurate information on a possible metabolic disease or risk factors involved in lithogenesis. Such metabolic investigation does not ensure the actual diagnosis of the lithogenic disease if stone composition does not match. Moreover, the stone composition during subsequent analysis can differ in up to 21 % of cases, implicating the necessity to send for morpho-constitutional analysis every different stone events/treatments [29].

Thus, in addition to metabolic investigation, stone analysis is an essential step for the etiological diagnosis. In some cases, the metabolic disease implied in stone formation is unrecognized by standard metabolic investigations while the stone may contain particular component allowing diagnosis unambiguously [12–15]. An example is adenine phosphoribosyltransferase deficiency revealed by a stone made of 2,8-dihydroxyadenine. To obtain this result, physical methods for stone analysis are required.

Stone analysis has to report qualitative and quantitative information regarding crystalline phases, their location within the stone and structural characteristics (morpho-constitutional analysis) [9, 20]. Such procedure consists in examining the surface and cross-sectional morphology to summarize stone features as a morphological type that can be related to metabolic disorders and diseases. Moreover, determining accurately the composition of all parts of the stone (the core, inner layers, peripheral layers and surface) is essential. Finally, a global qualitative analysis from the whole stone (or a fragment of the stone) with the relative proportions of all components identified by sequential analysis is recommended.

**Fig. 6:**
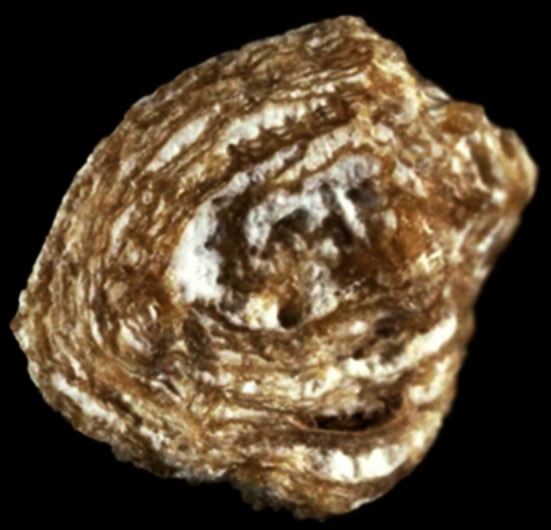
Type IIa + IVa mixed stone made of alternate layers of weddellite and carbapatite. Such a morphology and composition is highly suggestive of hypercalciuria. Of note, more than 50 % of calcium stones related to primary hyperparathyroidism exhibit such a structure

Morphological characteristics and the corresponding morphological types have been already described [9, 20]. In Table 1, the various types of stones are summarized, their corresponding main crystalline phase and the common causes associated with each stone subtype. About 98 % of urinary calculi are incorporated according to that classification. If a stone cannot be classified, two explanations should be considered: the chemical composition (e.g., dihydroxyadenine, xanthine, atazanavir, sulfadiazine…) or the cause is very uncommon [30]. Because calculi are frequently made of several crystalline phases, it is not surprising to find a mixture of different subtypes related to the different crystalline phases. Among the principal associations, finding binary mixtures such as COM and COD or ternary mixtures including COM, COD and carbapatite are counting together for a quarter of cases in our experience (Fig. 6).

**Table 1:** Main relations observed between stone type, main component and etiology

Morphological type	Subtype	Main components	Common causes
I	Ia	Whewellite	Dietary hyperoxaluria
	Ib	Whewellite	Stasis, low diuresis
	Ic	Whewellite	Primary hyperoxaluria type I
	Id	Whewellite	Malformative uropathy, stasis and confined multiple stones
	Ie	Whewellite	Enteric hyperoxaluria
II	IIa	Weddellite	Hypercalciuria
	IIb	Weddellite ± whewellite	Hypercalciuria ± hyperoxaluria ± hypocitraturia
	IIc	Weddellite	Hypercalciuria, stasis and confined multiple stones
III	IIIa	Uric acids	Low urine pH and stasis
	IIIb	Uric acids	Metabolic syndrome, diabetes
	IIIc	Various urates	Hyperuricosuria and alkaline urine, UTI
	IIId	Ammonium urate	Hyperuricosuria and diarrhea
IV	IVa1	Carbapatite	Hypercalciuria, UTI
	IVa2	Carbapatite	Distal renal tubular acidosis
	IVb	Carbapatite	UTI, hypercalciuria. Etiology depends on minor components identified in the stone
	IVc	Struvite	UTI by urease-splitting bacteria
	IVd	Brushite	Hypercalciuria, PHPT, phosphate leak
V	Va	Cystine	Cystinuria
	Vb	Cystine	Cystinuria + inadequate therapy
VI	VIa	Proteins	Chronic pyelonephritis
	VIb	Proteins	Proteinuria, drugs, clots
	VIc	Proteins	ESRF and excessive calcium + vitamin D supplementation

Main associations		
Ia or Ib + IIa or IIb	Whewellite + weddellite	Intermittent hyperoxaluria and hypercalciuria (dietary origin)
Ia + IVa1	Whewellite + carbapatite	Randall’s plaque, medullary sponge kidney
IIa or IIb + IVa1	Weddellite + carbapatite	Absorptive or resorptive hypercalciuria
Ia or Ib + IIa or IIb + IVa or IVb	Whewellite + weddellite + carbapatite	Hyperoxaluria + hypercalciuria, medullary sponge kidney
Ia + IIIb	Whewellite + uric acid	Hyperoxaluria + metabolic syndrome

*UTI* urinary tract infection, *PHPT* primary hyperparathyroidism, *ESRF* end-stage renal failure

The main limitation for an accurate stone analysis is the progress in flexible ureteroscopy with laser energy used to fragment a significant part of the stone, thus providing only small piece(s) for analysis. However, remembering that 60–80 % of urinary calculi pass spontaneously, they should be submitted to a morpho-constitutional analysis when collected.

## Keys for interpretation of stone analysis

### Qualitative aspects

The qualitative composition is imperative, and non-calcium-containing components are clinically relevant. Among purines:

Uric acid can be a marker for an excessive urate excretion in urine (hyperuricosuria), but it is more often related to insulin resistance with a defect in renal ammonia genesis and constant acidic urine. In less cases, it is related to a high urine concentration of urate because of low diuresis in patients treated a long time with uricosuric drugs or suffering inflammatory bowel disease that have required partial or total colectomy (digestive alkaline losses).

Uric acid may be identified as three different crystalline forms: anhydrous, monohydrate or dihydrate. There is no apparent difference between these three crystalline phases regarding the cause of the stone. However, uric acid dihydrate is the commonest crystalline phase of uric acid identified in crystalluria studies, and this phase is poorly stable with time, thus spontaneously converted to uric acid anhydrous after several weeks or months. Identifying uric acid dihydrate in a stone, especially as the principal form of uric acid, is highly suggestive of an active lithogenic process with recent stone formation.

In contrast, urate stones, irrespective to the cation that is linked to the urate anion, are always associated with an excessive urate concentration in urine because of low diuresis or high excretion of uric acid. Among the various urates reported, ammonium hydrogen urate and sodium hydrogen urate monohydrate are more frequent due to their relatively poor solubility. They are mainly found in alkaline urine. Sodium urate is seen in cases of high urate and high sodium concentration, while ammonium urate is observed with high ammonium concentration. The origin of ammonia is an overproduction by kidney cells as a compensatory mechanism of metabolic acidosis or a hydrolysis of urea by urea-splitting bacteria.

Dihydroxyadenine is a specific marker of an adenine phosphoribosyltransferase deficiency, a severe and rare genetic disease able to induce crystalline nephropathy and end-stage renal failure.

Xanthine suggests an overproduction of xanthine and/or a defect on the oxidative pathway to convert it into uric acid. Two pathological conditions may be involved: an inherited deficiency in xanthine dehydrogenase (familial xanthinuria) and an enzyme inhibition by allopurinol therapy in patients suffering an accelerated purine metabolism pathway as in Lesh–Nyhan syndrome (hypoxanthine–guanine phosphoribosyltransferase deficiency).

Methyl-1 uric acid is one of the primary metabolites of caffeine. It is produced in the liver by enzymes of the cytochrome P450. For a not clearly understood reason, methyl-1 uric acid was only identified in patients with increased blood aluminum level because of chronic ingestion of aluminum-containing drugs. It should alert the physician on potential aluminum toxicity.

Other components without calcium can crystallize in the urine: cystine, struvite, other magnesium phosphates, proteins and various drugs.

Cystine is a marker of cystinuria, which is the most common inherited tubular defect inducing stone formation.

Struvite is an indicator of urinary tract infection by urea-splitting micro-organisms. Other magnesium salts, without ammonium ion, namely newberyite and trimagnesium phosphate pentahydrate may be considered as struvite derivatives related to past UTI.

Proteins are present in all stones in slight proportion, commonly <5 %. In case they are more abundant, they must turn to particular causes of urolithiasis such as chronic pyelonephritis, severe chronic kidney disease, long-term treatment with some antiseptic or antiviral drugs. Among drugs able to form urinary stones, triamterene, atazanavir, sulfadiazine and ceftriaxone are the commonest. Ceftriaxone is identified in urinary or biliary tract as a calcium salt.

Regarding calcium oxalates and calcium phosphates, other criteria than the presence within the stone must be taking into account since these compounds are poorly soluble in urine. It is common to identify a small amount of carbapatite in the core of a calcium oxalate stone. The presence of carbapatite is of concern because it may be considered as the initiation process of the stone. It comes from Randall’s plaque, a papillary calcification that serves as a nidus of a growing number of CaOx stones in industrialized countries [31–34].

In our experience, the proportion of stones exhibiting a papillary print (umbilicated) was high among COM stones (39.6 %) and low (8.6 %) among COD calculi (*p* < 0.0001). Randall’s plaque among all spontaneously expulsed COM and COD stones were present in 60 and 16 %, respectively. Few data are available in other countries regarding the existence of Randall’s plaque identified from stone analysis. For example, in Spain [35] and Balearic Islands [36], Randall’s plaque was identified from stone examination in about 12.5 % of cases. Of note, several reports based on ureteroscopic examination of the renal papillae of stone formers underlined the high occurrence of Randall’s plaques in the kidneys, varying from 57 % in France [34] to 75–80 % in the USA [33, 37].

### Quantitative data

Most reports on stone composition in the literature focus on the principal component. It is a simple and useful approach from an epidemiological point of view. However, it is important to consider qualitative and quantitative aspects of the stone structure (including minor components) and to know the distribution of these elements within the stone (and the corresponding morphology).

#### Calcium oxalate

CaOx represents the main chemical species of stones throughout the world, and it can be identified as three different crystalline phases: COM, the most common; COD, frequency depending on the countries; and calcium oxalate trihydrate (COT), named caoxite, a rare and unstable phase. The comparison between urine biochemistry and crystalline phase of CaOx found in freshly voided urine provided evidence that COM crystals are related to hyperoxaluria, while COD crystals are mainly related to hypercalciuria [21, 38–40]. Thus, COM stones are mainly associated with excessive oxalate concentration (low diuresis) and/or excessive oxalate excretion with secondary mild and intermittent hyperoxaluria in 88 % of cases [38, 39, 41]. By contrast, COD stones are related to hypercalciuria in more than 85 % of cases [17]. The third phase (COT) is an infrequent and unstable form of CaOx and is observed in uncommon conditions including hyperoxaluria and specific drug intake [30, 42].

Among CaOx stones, a high proportion of them contains a mixture of COM and COD, often associated with carbapatite in various proportions (Randall’s plaque excluded). In such cases, biochemical factors involved in stone formation are those involved for each crystalline phase, i.e., hypercalciuria and hyperoxaluria. If an increased content of carbapatite is present, it should orient to more specific metabolic dysfunctions such as bone resorption, primary hyperparathyroidism, acidification tubular defect or another alkalinizing source.

#### Calcium phosphate

CaPh is a common chemical component of stones identified in about 85 % of all calculi in our experience (proportion: 0.5 up to 99 %). The clinical significance depends on the crystalline phase, the location and the overall content within the stone. Previously was underlined the increasing part of carbapatite Randall’s plaque as a nidus for CaOx stones. In such cases, the CaPh content is low (<5 %; Fig. 1). As suggested by Miller and coworkers, other CaOx stones which contain a core of carbapatite without papillary print may also result from an initiation on Randall’s plaque with a secondary coverage of the plaque by new layers of CaOx after the stone was unhooked from the papilla [43]. However, other causes of CaPh should be considered. Because carbapatite is highly pH dependent, it must be expected that carbapatite-rich stones are developed in poorly acidic to alkaline urine. This is suggestive for either urinary tract infection (UTI) or metabolic disorders responsible for chronically elevated urine pH, associated or not with hypercalciuria [44, 45]. Carbapatite associated with brushite and/or octacalcium phosphate pentahydrate (OCPP) is commonly a marker of hypercalciuria. Presence of OCPP indicates an active and recent lithogenic process [18].

UTI is one of the most common mechanisms resulting in phosphate stones [17, 44]. In such cases, several characteristics of the stone should be considered:

1. In addition to carbapatite, other CaPh species are present in the stone, in particular amorphous carbonated calcium phosphate and/or whitlockite.
2. A specific sign of UTI-induced calculi is the presence of struvite.
3. In the absence of struvite, another sign may be useful, namely the carbonation rate of carbapatite as determined by infrared spectroscopy [46]. When it is higher than 15 %, the probability that UTI is a driving force for stone formation is very high.

Carbapatite is rarely pure, and other crystalline phases present as minor components may assist to establish the diagnosis. Carbapatite associated with COD is highly suggestive of hypercalciuria and should raise suspicion for primary hyperparathyroidism [47]. In contrast, carbapatite associated with COM is more related to medullary sponge kidney and other causes of urinary stasis.

**Fig. 7:**
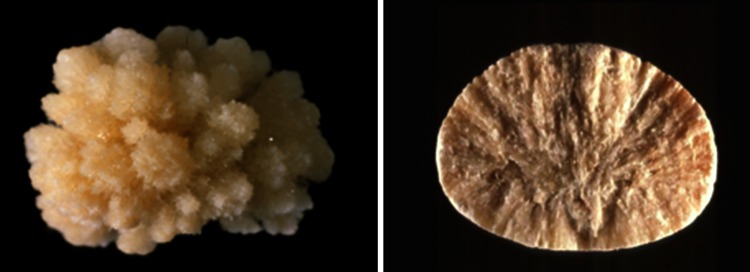
Examples of brushite stones type IVd (*left* surface; *right* section)

Among metabolic causes of carbapatite-rich stones, hypercalciuria is an important factor, the mechanism of which being often resorptive and/or absorptive [44, 48, 49]. Primary hyperparathyroidism is one of the main causes of resorptive hypercalciuria. Kidney stones related to such a pathological condition were recently considered, revealing particular features in stone composition and morphology (Figs. 6, 7) [47].

**Fig. 8:**
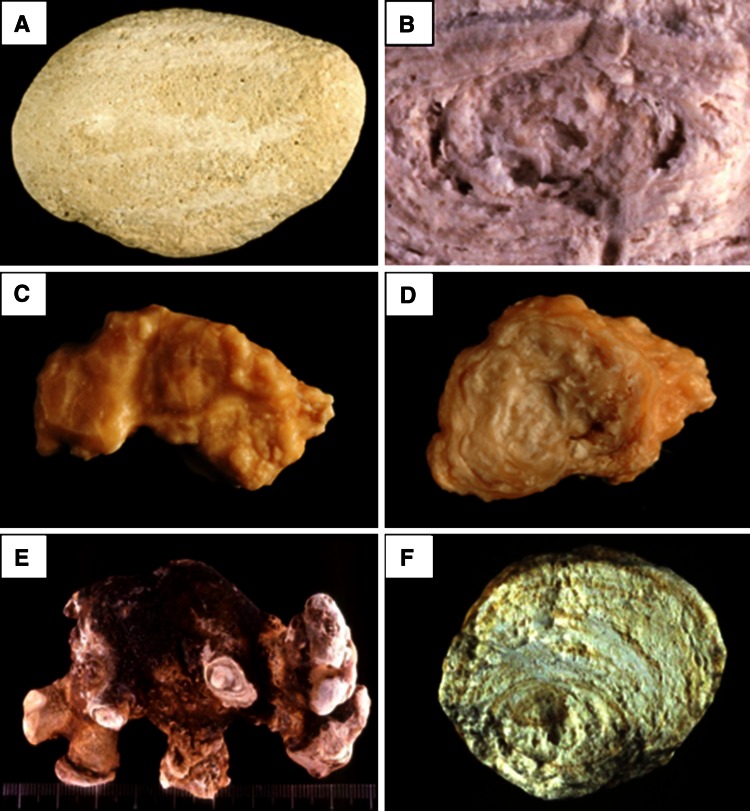
Calcium phosphate stones mainly composed of carbapatite: **a** subtype IVa1 (surface); **b** subtype IVa1 (section); **c** subtype IVa2 (surface): note the glazed aspect and the presence of very tiny cracks; **d** subtype IVa2 (section); **e** subtype IVb (surface); **f** subtype IVb (section)

An infrequent cause of CaPh stones is a kidney cells impairment to excrete protons as observed in inherited distal acidification defect or acquired auto-immune diseases such as Sjogren’s syndrome. In such metabolic diseases, urinary calculi are mainly composed of carbapatite with a very high content of CaPh, often above 80 % of the stone mass. While most causes of carbapatite stones induce IVa1 or IVb subtype, distal acidification defects are associated with IVa2 subtype in 90 % of cases (Fig. 8) [9]. Such findings illustrate the significance of morpho-constitutional analysis helping to find clinical diagnosis.

## The significant contribution of the stone morphology

### COM stones

COM accounts in most countries over the world as the more common and more abundant component of stones [50–53]. The morphological aspect of COM stones orients toward very different diseases or lithogenic conditions:

- Mild intermittent hyperoxaluria related to high oxalate intake
- Low diuresis with increased concentration of oxalate ions in urine
- Heavy hyperoxaluria either related to inherited diseases (primary hyperoxaluria type 1) or to enteric hyperoxaluria (ileal resection, bariatric surgery or chronic pancreatitis).

COM stones exhibit five different morphologies in class I of the morpho-constitutional classification.

**Fig. 9:**
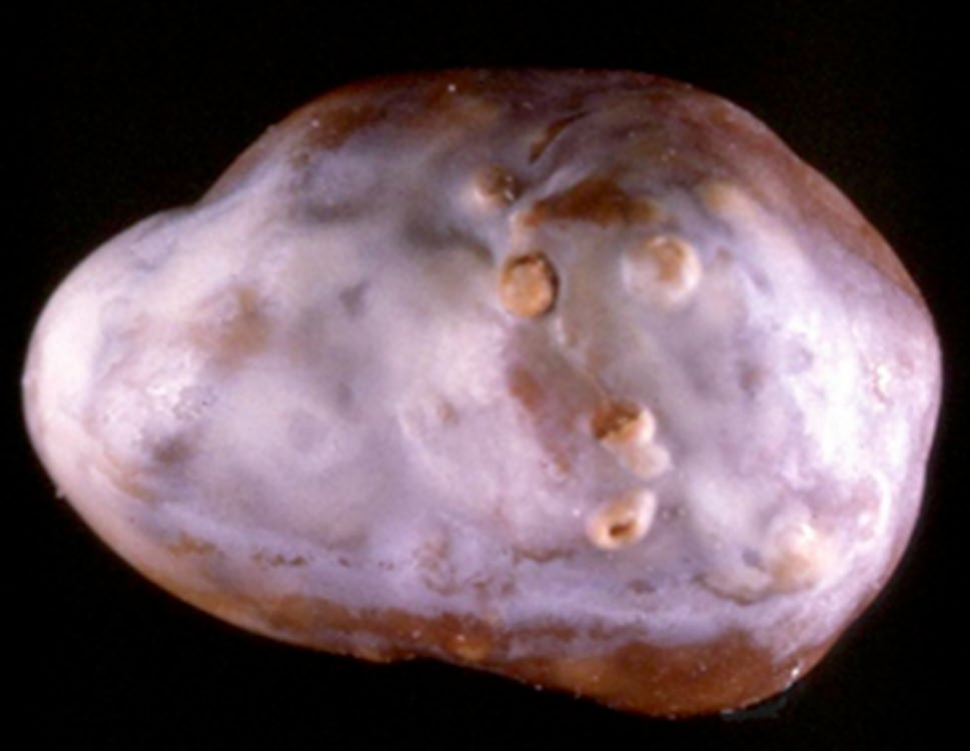
COM stone subtype Ia. Note the thin *grayish* layer of very recently deposited crystals covering the *brown* surface of the stone. Such a *grayish* coverage is resulting from recent episode of hyperoxaluria often related to transient oxalate-rich food intake

The subtype Ia (Fig. 1), often dark brown in color, suggests a slow and intermittent growth related to peaks of hyperoxaluria (low diuresis or oxalate-rich food intake). It is the most common subtype of calcium stones in most countries (unpublished data). While seeing a grayish thin layer on a Ia stone surface, it corresponds to a freshly COM crystal sediments secondary to a recent peak of urine concentration of oxalate (Fig. 9).

**Fig. 10:**
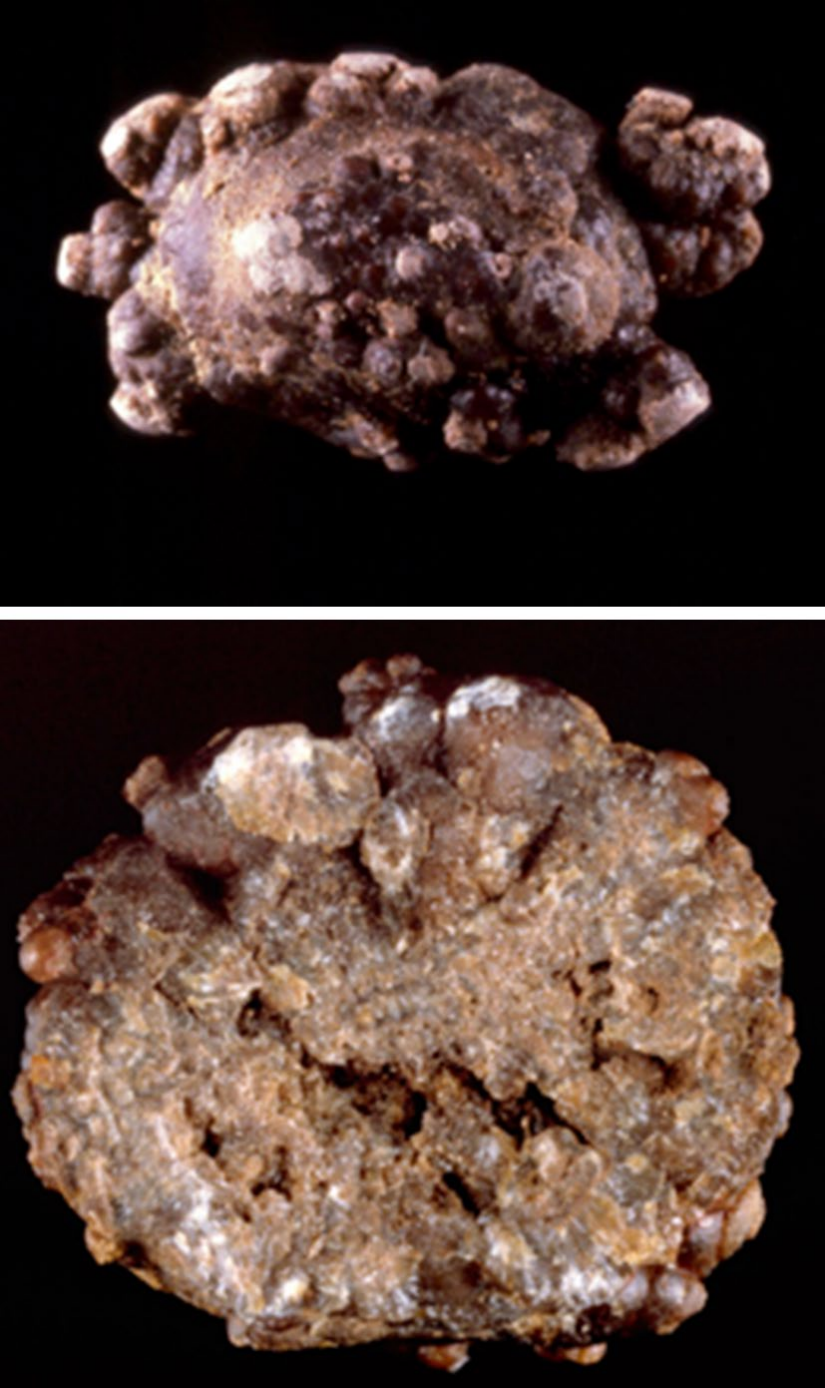
COM stones subtype Ib. *Top* surface, *bottom* section. Note the *dark color* of the stone in most parts of surface and section

The subtype Ib (Fig. 10) can be a marker of an old stone, probably first developed as weddellite because of transient hypercalciuria and secondly completely converted from weddellite to whewellite in the time. Subtypes Ia and Ib are often dark brown in color.

**Fig. 11:**
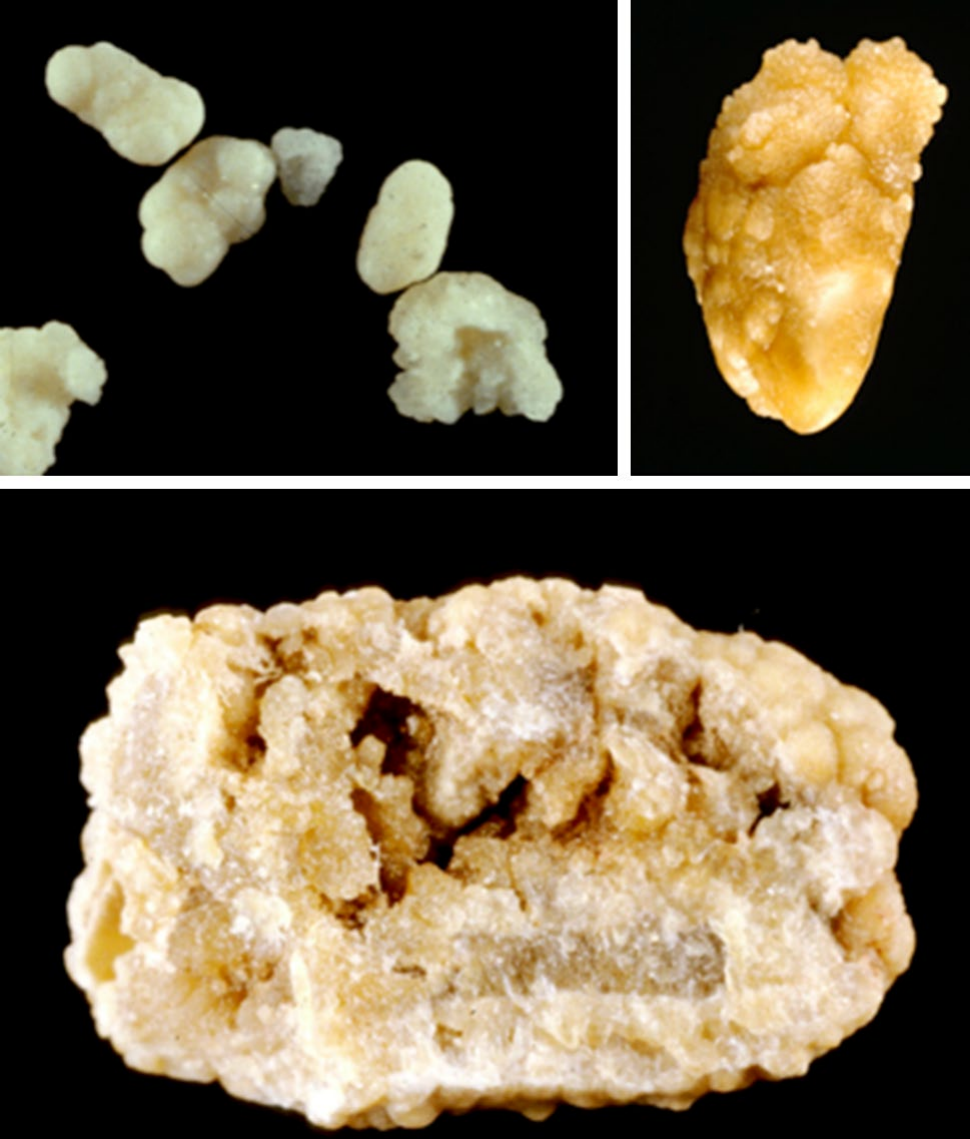
COM stones subtype Ic. *Top* surface. On the *left side*, the stones are whitish. They came from an infant aged <2 years. *Bottom* section. Note the* very light color* in most parts of the stones

In contrast, subtype Ic is very light, brown-yellow pale, or even white in children (Fig. 11). It is associated with heavy oxaluria, mainly primary hyperoxaluria type 1 (related to alanine glyoxylate aminotransferase deficiency in hepatocytes), which is the most severe stone disease often responsible for end-stage renal failure, especially when the diagnosis was delayed because stone morphology was not considered [54, 55]. All 92 stones from patients with PH type 1 analyzed in our laboratory had this Ic morphology, which appears to be virtually pathognomonic for the disease. Therefore, this particular morphology of pure COM stones should immediately orient the physician toward this severe disease to allow early introduction of proactive therapeutic strategy.

Other genetic forms of primary hyperoxaluria such as hyperoxaluria type 2 (glyoxylate reductase/hydroxypyruvate reductase deficiency) [56] or hyperoxaluria type 3 (related to a dysfunction of the 4-hydroxy 2-oxoglutarate aldolase in the hydroxyproline pathway) [57] do not present every time subtype Ic since hyperoxaluria is often associated with hypercalciuria for a not yet understood reason [58].

The subtype Id is typically a marker for stasis in patients with hyperoxaluria in a confined environment such as: calyceal diverticulum, ureteropelvic junction obstruction or prostate hypertrophy with incomplete bladder emptying (bladder stones).

**Fig. 12:**
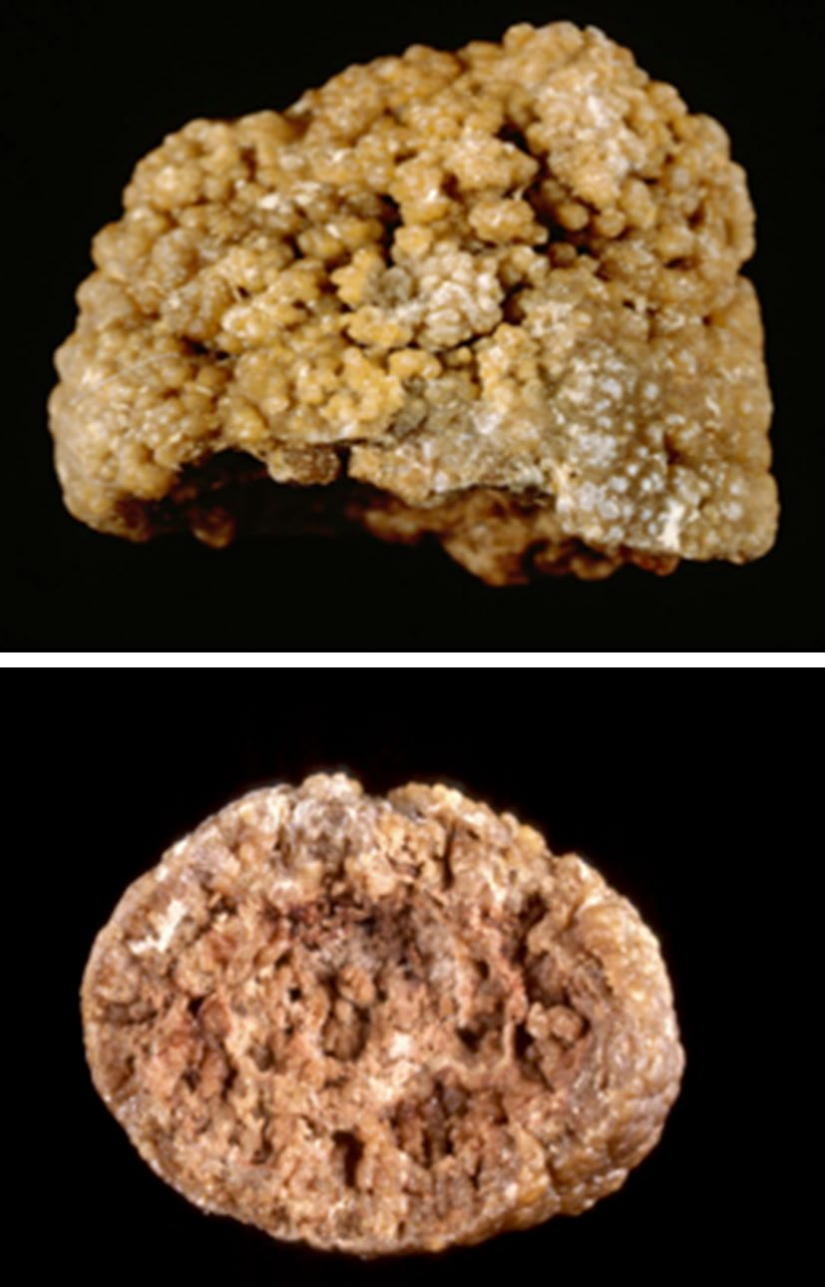
COM stones subtype Ie. *Top* surface, *bottom* section

Finally, the subtype Ie is related to severe forms of enteric hyperoxaluria in patients suffering from inflammatory bowel diseases with extensive ileal resections, bariatric surgery or chronic pancreatitis (Fig. 12) [9]. Among patients having enteric hyperoxaluria, subtype Ie was present in 82.5 % of cases.

Although Ic and Ie subtypes stones are scarce, it is important to identify these subtypes since they warn the clinician of a severe cause of hyperoxaluria, not always previously identified with investigations, and often responsible for a progressive kidney failure.

### COD stones

**Fig. 13:**
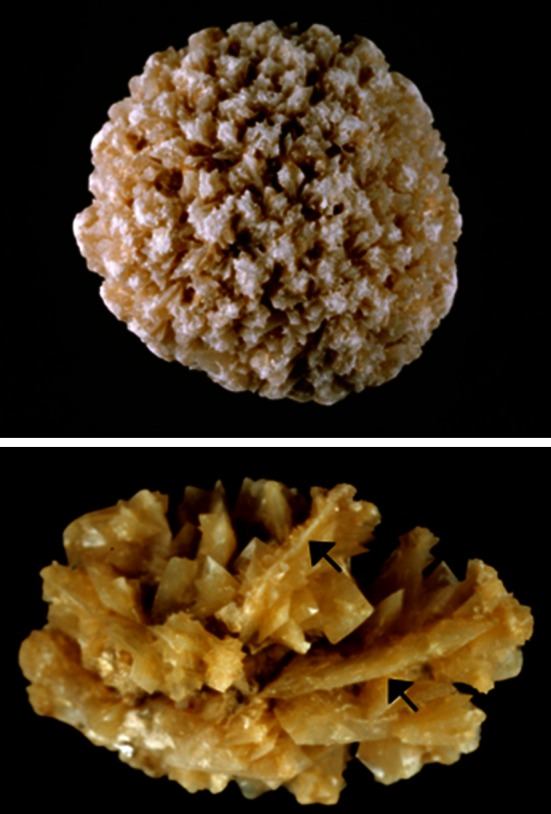
COD stones subtype IIa. *Top* stone made of small octahedral crystals of weddellite as commonly observed in patients who form stones because hypercalciuria. *Bottom* stone made of both small and very large crystals (*arrows*) of weddellite as commonly found in patients who suffered hypercalciuria, hyperoxaluria and, often mild hypocitraturia

Weddellite stones correspond to type II of the classification. Subtypes IIa to IIc are often related to hypercalciuria either associated or not with other conditions favoring stones growth. For example, we found that IIa or IIb subtypes made of large COD crystals were frequently related to hypercalciuria associated with hyperoxaluria and relative hypocitraturia (Fig. 13).

### Uric acid and urate stones

Remember that uric acid and urates account for type III of the classification and include four subtypes. Among them, subtypes IIIa and IIIb gather uric acid stones and subtypes IIIc and IIId gather urate stones that are commonly composed of ammonium hydrogen urate, which is the less soluble form of urate salts in urine.

**Fig. 14:**
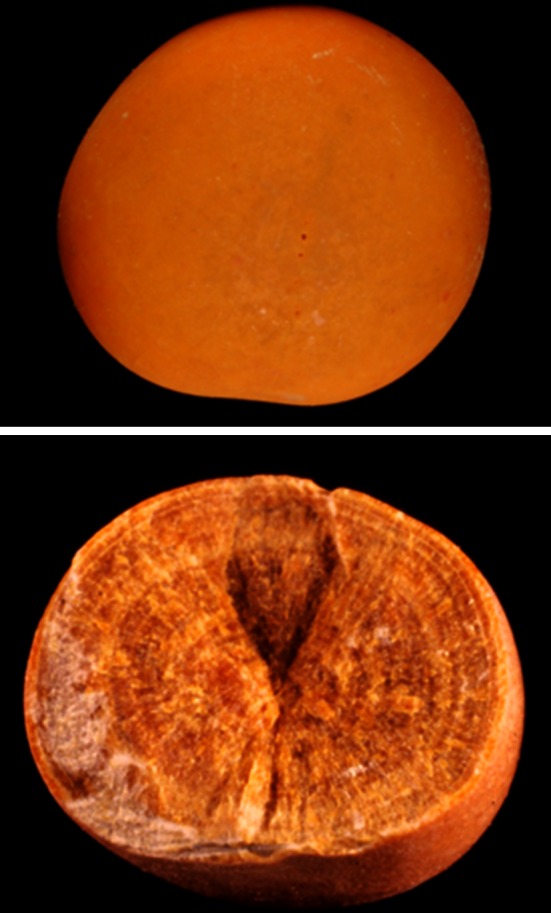
Uric acid stone subtype IIIa. *Top* surface, *bottom* section

Regarding uric acid stones, the subtype IIIa is primarily related to slow stone growth conditions as observed in urinary stasis and is mainly found with bladder stone of men with prostate hypertrophy (Fig. 14) By contrast, IIIb subtype suggests a substantial involvement of a metabolic process associated with one or several of the following factors:

- Permanent low urine pH in the case of metabolic syndrome or type 2 diabetes mellitus, or in the case of intestinal alkali loss in patients having chronic hydro-electrolytic diarrhea (ileostomy, colectomy, hemorrhagic rectocolitis,…)
- High excretion of uric acid as observed in diabetes mellitus, in myelo- or lymphoproliferative syndromes or the case of Vaquez disease or rare cases of tubular dysfunction inducing a defect in urate reabsorption. Of note, among patients suffering type 2 diabetes, females are especially at risk to develop uric acid stones exhibiting a subtype IIIb (37 vs. 13 % in the absence of diabetes, *p* < 0.00001).
- High production and excretion of uric acid from diet origin (high fructose intake, nucleo-protein rich food, rare genetic diseases on the nucleotide pathways such as Lesh–Nyhan syndrome or phosphoribosylpyrophosphate synthetase hyperactivity).
- High uric acid concentration in acidic and concentrated urine secondary to low diuresis, whatever the origin.

**Fig. 15:**
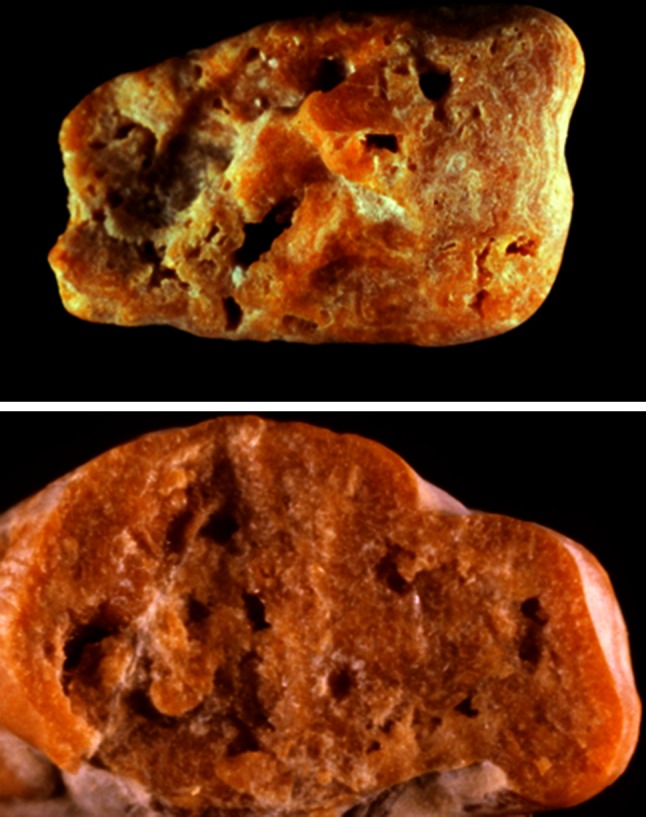
Uric acid stone subtype IIIb. *Top* surface, *bottom* section

In all cases, the high content of uric acid dihydrate in a IIIb stones is a marker of an active lithogenic process and a recent growth of the stone (Fig. 15).

Subtypes IIIc and IIId gather urate stones that have different etiologies than these observed for uric acid stones. In contrast with IIIa and IIIb stones, urate calculi are not developed in acidic urine and require a high urine concentration of urate in poorly acidic to alkaline urine. The main causes for urate stones are alkalizing conditions related to therapeutic measures or to urinary tract infection by urea-splitting micro-organisms or high urate concentration with low content of electrolytes in urine as found in chronic diarrhea. Among urate salts, ammonium urate is the most common component of IIIc subtype and the only one of IIId subtype.

The commonest form observed in Western countries corresponds to IIIc subtype calculi with a homogenous rough surface with local porous areas. The color is usually grayish. The inner structure is commonly loose, unorganized, and locally porous, the color being the same as in the surface. Such type of ammonium urate is found mainly in two pathological conditions:

- Local production of ammonium ions from urea in patients with UTI by urea-splitting bacteria;
- Excessive alkaline urine secondary to alkalinization for dissolving radiolucent stones (uric acid stone suspected) in patients with a preexisting hyperuricosuria.

The subtype IIId is seen among ammonium urate stones of children (mainly boys) with the endemic bladder lithiasis living in developing countries and anorectic patients living in industrialized countries. The inner structure of the stone is typical and appears as alternate concentric thick and thin layers, the former being compact and brownish, whereas the latter is loose and locally porous, beige in color. This stone subtype is perceived in cases of base loss due to chronic diarrhea with low phosphate intake, resulting in a compensatory increase in urinary ammonia excretion. The causes of diarrhea may be of infectious origin or laxative abuse in anorectic patients [59, 60]. Stone color observed with laxative abuse is very dark with purplish shades. In patients who have chronic diarrhea and ammonium urate stones, the subtype in more than 90 % of cases was IIId [9].

## Conclusion

Routine morpho-constitutional analysis of stones by morphologic examination combined with FTIR or X-ray diffraction considerably improves information from the stone analysis to determine the cause(s) of stone disease. It should be recommended in all laboratories that provide stone analysis for helping physicians to identify the causes of urolithiasis. It is remarkable that stones with the same chemical composition exhibit distinct morphological characteristics according to their cause, in relation with the degree of metabolic abnormalities and the kinetics of the lithogenic process.
